# Cardiometabolic disease among frailty phenotype clusters in adults aging with HIV

**DOI:** 10.1016/j.tjfa.2025.100011

**Published:** 2025-03-08

**Authors:** Raymond Jones, Ene M. Enogela, Stephanie A. Ruderman, Mari M. Kitahata, Richard Moore, Jeffrey M. Jacobson, Maile Karris, Meredith Greene, Julia Fleming, Sonia Napravnik, Greer Burkholder, Joseph A.C. Delaney, Heidi M. Crane, Amanda L. Willig, Thomas W. Buford

**Affiliations:** a University of Alabama at Birmingham, Birmingham, AL, USA; b University of Washington, Seattle, WA, USA; c Johns Hopkins University, Baltimore, MD, USA; d Case Western Reserve University, Cleveland, OH, USA; e University of California - San Diego, San Diego, CA, USA; f University of California – San Francisco, San Francisco, CA, USA; g Harvard Medical School, Fenway Institute, Boston, MA, USA; h University of North Carolina – Chapel Hill, Chapel Hill, NC, USA; i Birmingham/Atlanta Geriatric Research, Education, and Clinical Center, Birmingham VA Medical Center, Birmingham, AL, USA

**Keywords:** HIV, Frailty, Aging

## Abstract

**Background:**

Age-related morbidity, including frailty and cardiometabolic disease has become increasingly prevalent among people living with HIV (PWH), and each frailty characteristic may, independently and synergistically, play a role in cardiometabolic disease.

**Objective:**

To evaluate the prevalence of unique frailty clusters and the prevalence ratios of cardiometabolic diseases within frailty clusters among a large diverse cohort of PWH in clinical care.

**Design:**

Cross-sectional analyses within longitudinal clinical cohort.

**Setting:**

The Center for AIDS Research Network of Integrated Clinical Systems (CNICS) from 8 Clinics

**Participants:**

4,856 PWH, mean age 61 years. 16 % frail, 45 % pre-frail, 40 % robust.

**Measurements:**

The validated, modified Fried Phenotype from patient-reported outcomes and clustering (15 clusters) of the frailty characteristics and cardiometabolic diseases (7 diseases and multimorbidity) within each cluster.

**Results:**

Among 4856 PWH (age: 61 ± 6 years), the prevalence of frail, pre-frail, and robust was 16 %, 45 %, and 40 %, respectively. The most prevalent cardiometabolic disease among frail PWH was hypertension (62.6 %), followed by dyslipidemia (58.8 %) and diabetes (31.4 %). Among pre-frail PWH, the most prevalent cardiometabolic diseases were dyslipidemia (65.8 %), hypertension (61.8 %), and obesity (30.5 %). The prevalence of cardiometabolic disease among frailty clusters varied. For example, PWH in the “fatigue + poor mobility” cluster had a greater prevalence of cerebrovascular disease (PR: 2.23; 95 % CI: 1.01–4.91), diabetes (1.76; 95 % CI: 1.41–2.21), and obesity (1.66; 95 % CI: 1.35–2.05) when compared with robust PWH. Individuals in the “poor mobility” cluster had a higher prevalence of diabetes (1.37; 95 % CI: 1.15–1.64), hypertension (1.12; 95 % CI: 1.04 – 1.22), and obesity (1.38; 95 % CI: 1.17–1.61) compared with robust PWH.

**Conclusions:**

The frailty components, independently and synergistically, were associated with an increased prevalence of cardiometabolic disease. This study identified distinct frailty clusters that may be associated with increased prevalence of cardiometabolic disease among PWH.

## 1 Introduction

HIV-related morbidity and mortality have declined with the advent of antiretroviral therapy (ART) [1]. This has resulted in an increase in age-related morbidity, including frailty and cardiometabolic disease, which has become prevalent among people living with HIV (PWH) [2]. Frailty describes the vulnerability of an individual to stressors and is associated with mortality and other adverse health outcomes, including falls and hospitalizations [3,4]. With accelerated and accentuated aging occurring in PWH, frailty may occur at earlier ages with the transition from pre-frailty to frailty occurring faster. Thus, a more intensive, low-burden assessment of frailty may facilitate successful aging among PWH through better ascertainment of frailty and associated risk for PWH in HIV care and other settings. The present study highlights this through validated self-report frailty measures that can be implemented into larger cohorts with less burden on participants. This assessment strategy could provide insight into potential strategies to slow the progression of frailty or potentially reverse the condition.

Given the significance of frailty in PWH and the shift from infectious disease complications to chronic illnesses, functional outcomes and frailty have become a focal domain among PWH. The most used assessment of frailty is the Fried frailty phenotype [5], which includes five components of physical health and functional status including low physical activity, exhaustion, weakness, slowness, and unintentional weight loss. This definition is well-recognized and has tremendous utility, however, identifying clustering of the frailty characteristics – consisting of a subgroup of the distinct characteristics co-occurring – may provide a better understanding of frailty risk among older PWH.

Reports on the prevalence of unique clustering of frailty in the context of health outcomes such as cardiometabolic disease remain scarce. Examining this concept of frailty characteristic clustering has the potential to capture distinct pathways of disease etiologies that affect functional independence and quality of life among PWH. While previous evidence has implicated various individual frailty characteristics in the deterioration of physical function and poor health outcomes in PWH (e.g., low grip strength associated with obesity; [6] low physical activity associated with CVD and diabetes; [7] etc.), these characteristics rarely occur in isolation. This is evidenced by Fried et al. [5] and Xue et al. [8] who identified many clinical underpinnings of the frailty characteristics and how the characteristics are linked, independently and synergistically.

Researchers with the Centers for AIDS Research Network of Integrated Clinical Systems (CNICS), a large U.S.-based cohort of PWH [9], developed and validated a modified Fried phenotype based on four self-reported components, including 1) unintentional weight loss, 2) low physical activity, 3) fatigue, and 4) poor mobility [10]. This modified phenotype is similar to others used in cohorts of PWH including the exclusion of weakness measured via grip strength, which is not collected at routine clinic visits in CNICS [11,12]. The four components of the modified Fried phenotype, used and validated within CNICS, have been observed as strong standalone predictors of poor outcomes among the general population and PWH; thus, it is important to evaluate the clustering of characteristics of the modified phenotype within CNICS to understand frailty risk and cardiometabolic outcomes among PWH.

Understanding the various clusters that exist in the modified phenotype and linking those clusters to the burden of cardiometabolic outcomes is likely to provide better risk stratification to potentially manage and/or reverse frailty. Therefore, the objective of this cross-sectional, observational study was twofold; we sought to 1) describe the prevalence of unique clusters; and 2) evaluate the prevalence ratios of frailty clusters with cardiometabolic disease in older PWH in the CNICS cohort.

## 2 Methods

### 2.1 Study population and setting

This study included PWH ages 50 years and older, who were in care in CNICS between 2011 and 2021 [9]. CNICS is a clinical cohort of PWH from 8 (expanding to 10) clinics across the United States, which integrates and harmonizes clinical data from electronic health records and other sources (e.g., demographic characteristics, diagnosis, laboratory and vital signs, medication, health care utilization, etc.) gathered during routine clinical visits [9]. Additionally, PWH in CNICS complete a clinical assessment of patient-reported outcomes (PROs), which consists of validated survey instruments, such as the HIV Symptom Index [13] and 9-item Patient Health Questionnaire [14]. These PROs are completed approximately every 4–6 months as part of routine clinic visits. Eligibility criteria for the current study include (1) aged ≥ 50 years, (2) prescribed antiretroviral therapy, (3) complete data from at least one visit on all frailty characteristics collected in CNICS, (4) complete data on demographics. Institutional review boards (IRB) of participating sites approved CNICS study protocols and secondary analysis of de-identified data, and participants completed informed consent before entry into CNICS. Ethical approval was provided by the IRB at The University of Alabama at Birmingham (IRB-300,009,373).

### 2.2 Frailty and pre-frailty

Frailty was defined using the modified Fried phenotype based on 4 components [5]. Included components were from the PRO assessments of fatigue (HIV Symptom Index), unintentional weight loss (HIV Symptom Index), poor mobility (EuroQOL Health-Related Quality of Life questionnaire), and low physical activity (Lipid Research Questionnaire) [3]. The component not included in Fried's original phenotype was muscle weakness, measured via grip strength, which is not assessed in CNICS. However, the modified Fried phenotype has good validation properties when compared with the Fried phenotype (ρ = 0.81) [3]. Each of the four available components were dichotomized and PWH were scored from 0 to 4 based on the presence of the components. Frailty status was considered with three levels: robust (0 components), pre-frail (1–2 components), or frail (≥3 components) [3,5].

### 2.3 Frailty clusters

Following the categorical assigning of PWH into robust, pre-frail, and frail, we used combinations to identify unique clusters of the frailty components within CNICS. The analytic combinations resulted in 15 clusters from 1-component (*4 clusters*: “fatigue”, “weight loss”, “low physical activity”, “poor mobility”); 2-component (*6 clusters*: “fatigue + weight loss”, “fatigue + low physical activity”, “fatigue + poor mobility”, “weight loss + low physical activity”, “weight loss + poor mobility”, “low physical activity + poor mobility”); 3-component (*4 clusters*: “fatigue + weight loss + low physical activity”, “weight loss + low physical activity + poor mobility”, “fatigue + low physical activity + poor mobility”, “fatigue + weight loss + slowness”); and 4-component (*1 cluster*: “fatigue + weight loss + low physical activity + poor mobility”). Clusters were mutually exclusive, such that PWH were included in only one cluster for analyses. The most recent patient reported frailty measure was adopted for all analyses in this study.

### 2.4 Cardiometabolic diseases

This study focused on eight cardiometabolic disease outcomes: cardiovascular diseases (CVD), hypertension, diabetes, cerebrovascular diseases, obesity, chronic kidney disease (CKD), dyslipidemia, and multimorbidity. 1) CVD was classified if PWH were diagnosed with a myocardial infarction, which was centrally adjudicated within CNICS by physician reviewers [15]. 2) Hypertension was considered present if participants had a diagnosis of hypertension and the presence of an antihypertensive medication. 3) Diabetes was defined as having either a hemoglobin A1c ≥ 6.5 % or the presence of a diabetes specific medication (e.g., insulin). For participants with diabetes-related medications that were not exclusively used for the management of diabetes alone, PWH must also have had a diabetes diagnosis in the electronic medical record [16]. 4) Cerebrovascular disease was defined as having clinical records of a cerebrovascular event. Cerebrovascular events (i.e., strokes) were ascertained from linkages with the electronic medical records as they had not all been adjudicated in CNICS during the time of this analysis. 5) Obesity (BMI ≥ 30 kg/m^2^ based on CDC definition) was ascertained using height and weight collected during routine clinic visits. 6) CKD was ascertained using confirmed diagnosis and an estimated glomerular filtration rate <60 mL/min/1.73 m2 for >3 months (2 values >90 days apart without an intervening normal value). 7) Dyslipidemia was considered present when participants were taking lipid-lowering medications, specifically statins. 8) Multimorbidity was defined as having 2 or more of the 7 diseases.

### 2.5 Statistical analysis

Patient characteristics were contrasted using chi-square tests and one-way analysis of variance (ANOVA). We compared demographic and clinical information across frailty (i.e., robust, pre-frail, frail). We examined the prevalence of frailty and pre-frailty clusters within the cohort. Additionally, we described the prevalence of cardiometabolic disease across frailty and by frailty clusters.

For cardiometabolic disease outcomes, relative risk regression models and the modified Poisson version [17] were used to compute prevalence ratio and 95 % confidence intervals (CI) for the presence of each cardiometabolic condition within the clusters (compared to the robust group, i.e. frail versus robust, or pre-frail versus robust) and controlling for demographic characteristics (e.g., age and sex assigned at birth). For all analyses, the robust group was the referent group. All tests were two-sided, and a p-value of < 0.05 was considered statistically significant for all analyses. All analyses were carried out using SAS version 9.4 (SAS Institute Inc., Cary, NC, USA).

## 3 Results

Of the ∼40,000 participants in CNICS, 6075 individuals were in care during the study period after the PRO assessment was initiated, and were 50 years of age or older and, and taking ARTs. After exclusion of missing data for any PRO assessment of frailty, 4856 PWH were included in the analyses for this study. Overall, the mean age was 61 ± 6.4 years for PWH included. The majority of PWH (62.2 %) self-reported White race and were male (83.8 %). The prevalence of frail, pre-frail, and robust was 16 %, 45 %, and 40 %, respectively. Additional demographic and clinical characteristics are shown in Table 1.

**Table 1:** Demographic and clinical characteristics by frailty status among people living with HIV who are ≥50 years old in clinical care across the United States.

	Total *N* = 4856	Frail *n* = 762	Pre-frail *n* = 2163	Robust *n* = 1931	p value
Characteristics					
Age| mean ± SD	61 ± 6.4	60 ± 6.5	61 ± 6.4	61 ± 6.3	0.004
Birth Sex					
Male	4071 (83.8 %)	615 (80.7 %)	1787 (82.6 %)	1669 (86.4 %)	0.0002
Female	785 (16.2 %)	147 (19.3 %)	376 (17.4 %)	262 (13.6 %)	
Race^a^					
White	3020 (62.2 %)	513 (67.3 %)	1313 (60.7 %)	1194 (61.8 %)	0.03
Black	1489 (30.7 %)	194 (25.5 %)	690 (31.9 %)	605 (31.3 %)	
Others	231 (4.8 %)	37 (4.9)	102 (4.7)	92 (4.8)	
Hispanic Ethnicity^b^	559 (12.7 %)	84 (11.8 %)	245 (12.3 %).	230 (13.5 %)	0.42
Transgender^c^					
Yes	37 (1.1 %)	12 (2.2 %)	16 (1.1 %)	9 (0.7 %)	0.02
No	3380 (98.9 %)	541 (97.8 %)	1482 (98.9 %)	1357 (99.3 %)	
Transmission risk factor					
IVDU	368 (7.6 %)	89 (11.7 %)	187 (8.7 %)	92 (4.8 %)	<0.0001
MSM	2855 (58.8 %)	416 (54.6 %)	1246 (57.6 %)	1193 (61.8 %)	
Heterosexual	1172 (24.1 %)	159 (20.9 %)	516 (23.9 %)	497 (25.7 %)	
IVDU + MSM	245 (5.1 %)	64 (8.4 %)	110 (5.1 %)	71 (3.7 %)	
Others/Unknown	216 (4.5 %)	34 (4.5 %)	104 (4.8 %)	78 (4.0 %)	
Clinical characteristics					
Blood pressure^d^					
SBP (mmHg)	128 (121 – 134)	128 (120 – 134)	128 (121 – 134)	128 (121 – 135)	0.12
DBP (mmHg)	79 (75 – 83)	78 (74 – 83)	79 (75 – 83)	80 (75 – 84)	<0.0001
CD4+ *T*-cell count (cells/μl)^e^	506 (343 – 701)	478 (315 – 688)	507 (342 – 718)	514 (358 – 691)	0.02
CD4/CD8 ratio^f^	0.59 (0.39 – 0.91)	0.55 (0.35 - 0.89)	0.59 (0.39 – 0.89)	0.62 (0.41 – 0.94)	0.001

IQR = Interquartile range; IVDU = Intravenous drug use; MSM = Men having sex with men; SBP = systolic blood pressure; DBP = diastolic blood pressure; mean ± SD: mean, standard deviation. All continuous measures are reported in median and interquartile range except where otherwise stated.

p-value: Continuous measures (mean and standard deviation) compared with an ANOVA or a Kruskal Wallis test (median and interquartile range), and a Pearson chi square test for categorical measures.

a Missing race *n* = 116; Other race include American Indian, Asian, Pacific islander, multiracial.

b Not reported for *n* = 460.

c Not reported for *n* = 1439.

d Median BP proportions missing in *n* = 65.

e Median CD4+ *T*-cell count missing in *n* = 5.

f Median CD4/CD8 ratio missing in *n* = 626.

### 3.1 Prevalence of clusters and cardiometabolic diseases

Among frail PWH, the most common cluster was “fatigue + poor mobility + low physical activity” (37.8 %), followed by the “fatigue + weight loss + poor mobility + low physical activity” cluster (24.4 %). The most frequent pre-frail clusters were poor mobility (19.1 %) and low physical activity (17.7 %). Prevalence for all clusters is shown in Table 2.

**Table 2:** Unique frail and pre-frail clusters among people living with HIV in the CNICS cohort.

Frailty categories, n (%)		
Frail		*N* = 762
	Fatigue + Poor mobility + Low Physical Activity	288 (37.8)
	Fatigue + Weight Loss + Poor mobility + Low Physical Activity	186 (24.4)
	Weight Loss + Poor mobility + Low Physical Activity	106 (13.9)
	Fatigue + Weight Loss + Poor mobility	102 (13.4)
	Fatigue + Weight Loss + Low Physical Activity	80 (10.5)
Pre-frail		*N* = 2163
	Poor mobility	412 (19.1)
	Low Physical Activity	382 (17.7)
	Poor mobility + Low Physical Activity	302 (14.0)
	Fatigue	262 (12.1)
	Weight Loss	223 (10.3)
	Fatigue + Poor mobility	166 (7.7)
	Fatigue + Low Physical Activity	144 (6.7)
	Weight Loss + Poor mobility	108 (5.0)
	Fatigue + Weight Loss	88 (4.1)
	Weight Loss + Low Physical Activity	76 (3.5)

Among frail PWH (Table 3), the most prevalent cardiometabolic disease was hypertension (62.6 %), followed by dyslipidemia (58.8 %) and diabetes (31.4 %). Additionally, 36.9 % of the frail group had multimorbidity (≥2 diseases). Among PWH who were pre-frail, the most prevalent cardiometabolic diseases were hypertension (61.8 %), dyslipidemia (65.8 %), and obesity (30.5 %). Comparable to the frail group, multimorbidity was present in 38.8 % of the pre-frail group, while 29.8 % of robust PWH had multimorbidity.

**Table 3:** Prevalence of cardiometabolic diseases by frailty status among PWH in the CNICS cohort.

	Overall *N* = 4856	Frail *n* = 762	Pre-frail *n* = 2163	Robust *n* = 1931	P value
Cerebrovascular disease	129 (2.7)	23 (3.0)	70 (3.2)	36 (1.9)	0.02
Cardiovascular Disease	330 (6.8)	73 (9.6)	163 (7.5)	94 (4.9)	<0.0001
Diabetes Mellitus	1215 (25.0)	239 (31.4)	600 (27.7)	376 (19.5)	<0.0001
Chronic Kidney disease*	948 (19.5)	147 (19.3)	432 (20.0)	369 (19.1)	0.78
Hypertension	2877 (59.3)	477 (62.6)	1336 (61.8)	1064 (55.1)	<0.0001
Dyslipidemia	3078 (63.4)	448 (58.8)	1424 (65.8)	1206 62.5)	0.001
Obesity*	1251 (26.9)	197 (27.1)	633 (30.5)	421 (22.7)	<0.0001
Multimorbidity (> 2 CMDs)	1695 (34.9)	281 (36.9)	839 (38.8)	575 (29.8)	<0.0001

⁎ Obesity missing *n* = 196; CKD missing *n* = 3; p-value: Pearson chi square.

Table 4 shows the prevalence of cardiometabolic diseases within the clusters. Among frail PWH, the most prevalent cardiometabolic diseases were recorded among those in the cluster of “fatigue + poor mobility + low physical activity” (obesity: 49.2 %; diabetes: 40.6 %; dyslipidemia: 40.2 %; CKD: 40.0 %; hypertension: 39.4 %; cerebrovascular disease: 34.8 %; CVD: 34.3 %). Notably, among pre-frail PWH, individuals in the “poor mobility + low physical activity” cluster had cerebrovascular disease: 25.7 %; CVD: 24.5 %; diabetes: 20.2 %; CKD: 18.7 %; obesity: 16.4 %; hypertension: 16.4 %; and dyslipidemia: 15.2 %.

**Table 4:** Prevalence of cardiometabolic diseases by unique frailty phenotypic clusters.

		Cerebrovascular disease	Cardiovascular disease	Diabetes mellitus	Chronic kidney disease^a^	Hypertension	Dyslipidemia	Obesity^b^
Pre-Frail, *n* = 2163		70	163	600	432	1336	1424	633
	Fatigue + Weight Loss	2 (2.9)	3 (1.8)	12 (2.0)	11 (2.6)	45 (3.4)	49 (3.4)	12 (1.9)
	Fatigue + Poor mobility	7 (10.0)	7 (4.3)	58 (9.7)	26 (6.0)	101 (7.6)	109 (7.7)	61 (9.6)
	Fatigue + Low Physical Activity	–	11 (6.8)	44 (7.3)	32 (5.3)	82 (6.1)	96 (6.7)	54 (8.5)
	Weight Loss + Poor mobility	5 (7.1)	15 (9.2)	33 (5.5)	23 (5.3)	76 (5.7)	69 (4.9)	29 (4.6)
	Weight Loss + Low Physical Activity	1 (1.4)	3 (1.8)	20 (3.3)	13 (3.0)	39 (2.9)	46 (3.2)	21 (3.3)
	Poor mobility + Low Physical Activity	18 (25.7)	40 (24.5)	121 (20.2)	71 (16.4)	219 (16.4)	216 (15.2)	104 (16.4)
	Fatigue	7 (10.0)	20 (12.3)	55 (9.2)	50 (11.6)	146 (10.9)	175 (12.3)	61 (9.6)
	Weight Loss	5 (7.1)	15 (9.2)	42 (7.0)	39 (9.0)	117 (8.8)	134 (9.4)	47 (7.4)
	Poor mobility	14 (20.0)	26 (16.0)	116 (19.3)	83 (19.2)	266 (19.9)	270 (19.0)	130 (20.5)
	Low Physical Activity	11 (15.7)	23 (14.1)	99 (16.5)	84 (19.4)	245 (18.3)	260 (18.3)	114 (18.0)
Frail, n (%)		23	73	239	147	477	448	197
	Fatigue + Weight Loss + Poor mobility + Low Physical Activity	4 (17.4)	23 (31.5)	58 (24.3)	45 (30.6)	122 (25.6)	109 (24.3)	42 (21.3)
	Fatigue + Weight Loss + Poor mobility	3 (13.0)	4 (5.5)	30 (12.6)	17 (11.6)	54 (11.3)	46 (10.3)	19 (9.6)
	Fatigue + Weight Loss + Low Physical Activity	2 (8.7)	10 (13.7)	21 (8.8)	9 (6.1)	47 (9.9)	51 (11.4)	13 (6.6)
	Fatigue + Poor mobility + Low Physical Activity	8 (34.8)	25 (34.3)	97 (40.6)	55 (37.4)	188 (39.4)	180 (40.2)	97 (49.2)
	Weight Loss + Poor mobility + Low Physical Activity	6 (26.1)	11 (15.8)	33 (13.8)	21 (14.3)	66 (13.8)	62 (13.8)	26 (13.2)

a Missing *n* = 3.

b Missing *n* = 196.

### 3.2 Prevalence of cardiometabolic diseases by pre-frailty clusters (Fig. 1; supplemental Table 1)

**Fig. 1:**
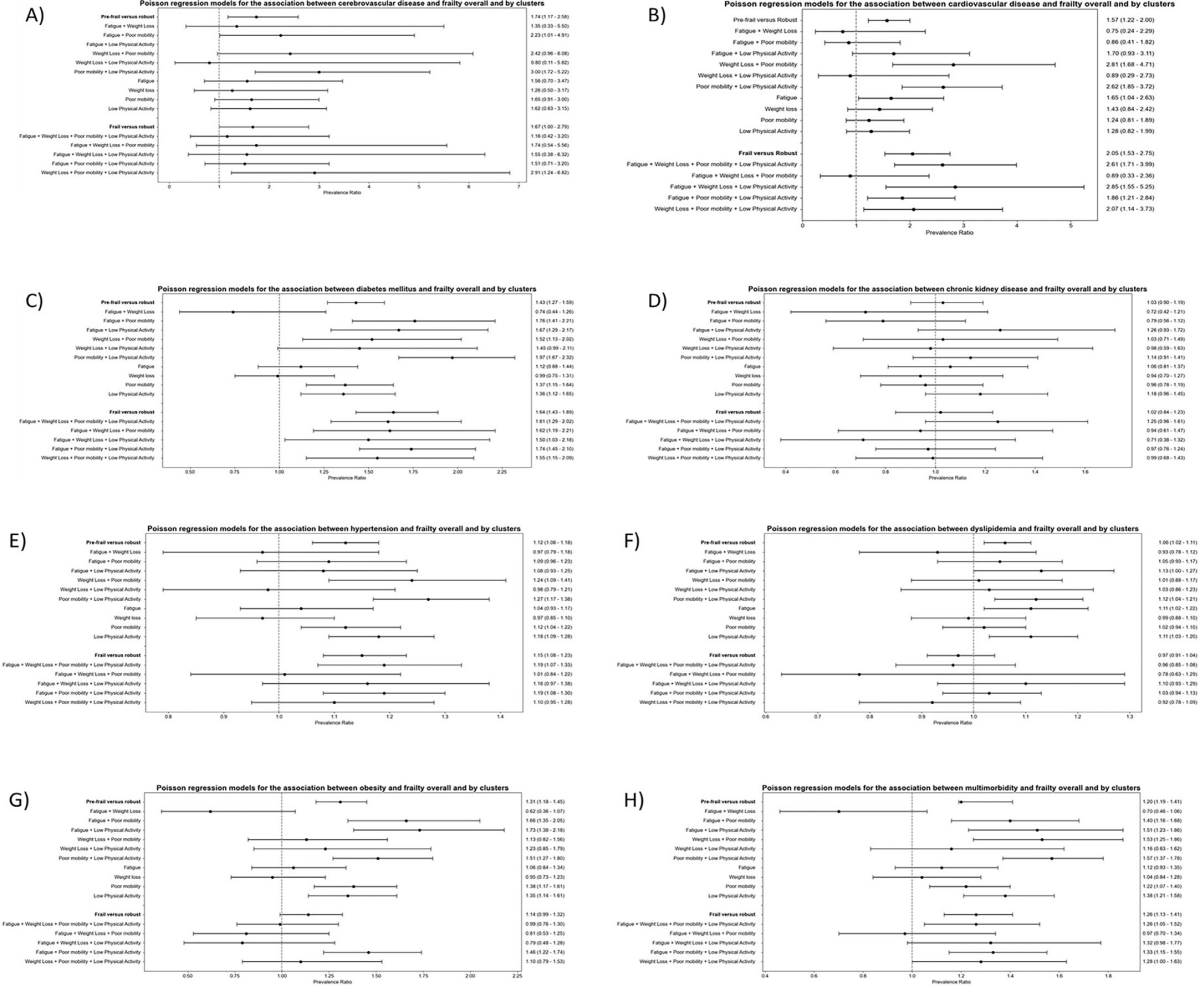
Forest plots showing associations between frailty clusters and individual cardiometabolic conditions. From Left to Right: a: Cerebrovascular Disease; b: Cardiovascular Disease; c: Diabetes Mellitus; d: Chronic Kidney Disease; e: Hypertension; f: Dyslipidemia; g: Obesity; h: Multimorbidity.

Overall, PWH who were classified as pre-frail had a greater age and sex adjusted prevalence of cerebrovascular disease (PR: 1.74; 95 %CI: 1.17 – 2.58), CVD (PR: 1.57; 95 %CI: 1.22 – 2.00), diabetes (PR: 1.43; 95 %CI: 1.27 – 1.59), hypertension (PR: 1.12; 95 %CI: 1.06 – 1.18), dyslipidemia (PR: 1.06; 95 %CI: 1.02 – 1.11), and obesity (PR: 1.31; 95 %CI: 1.18 – 1.45) compared with robust. In the most prevalent 1-component cluster of “poor mobility”, PWH had higher prevalence of diabetes (PR: 1.37; 95 % CI: 1.15 – 1.64), hypertension (PR: 1.12; 95 % CI: 1.04 – 1.22), and obesity (PR: 1.38; 95 % CI: 1.17 – 1.61) compared with the robust PWH. PWH in the most prevalent 2-component cluster of “poor mobility + low physical activity” had higher prevalence of all cardiometabolic diseases, except CKD. Additional prevalence ratios for remaining pre-frailty clusters are depicted in Fig. 1 and **Supplemental Table 1**.

### 3.3 Prevalence of cardiometabolic diseases by frailty clusters (Fig. 1 & supplemental Table 1)

Overall, PWH who were classified as frail had a greater age and sex adjusted prevalence of CVD (PR: 2.05; 95 %CI: 1.53 – 2.75), diabetes (PR: 1.64; 95 %CI: 1.43 – 1.89), and hypertension (PR: 1.15; 95 %CI: 1.08 – 1.23) compared with robust. PWH in the most prevalent frailty cluster “fatigue + poor mobility + low physical activity” cluster, there was a greater prevalence of CVD (PR: 1.86; 95 %CI: 1.21 - 2.84), diabetes (PR: 1.74; 95 %CI: 1.45 - 2.10), hypertension (PR: 1.19; 95 %CI: 1.08 - 1.30), and obesity (PR: 1.46; 95 %CI: 1.22 - 1.74). Lastly, PWH in the “fatigue + weight loss + poor mobility + low physical activity” frailty cluster had higher prevalence of CVD (PR: 2.61; 95 %CI: 1.71 – 3.99), diabetes (PR: 1.61; 95 %CI: 1.29– 2.02), and hypertension (PR: 1.19; 95 %CI: 1.07 – 1.33) compared with robust PWH. Prevalence ratios are shown in Fig. 1 and **Supplemental Table 1** for the remaining frailty clusters.

The prevalence of multimorbidity among the frailty and pre-frailty clusters are shown in Fig. 1 **(& Supplemental Table 2)**.

**Supplemental Table 3** and **Supplemental Table 4** show the prevalence of cardiometabolic diseases among clusters after adjustment for demographics and HIV-related factors (e.g., CD4 cell count, ART status). There was no difference in prevalence of cardiometabolic within the frailty clusters in this model when compared with the demographic-only adjusted model.

## 4 Discussion

We evaluated the prevalence of unique frailty clusters and cardiometabolic outcomes among PWH ≥ 50 years old in CNICS, a multisite cohort of PWH in clinical care across the US. We found that PWH who were pre-frail had greater prevalence of hypertension, dyslipidemia, and obesity, while PWH who were frail had greater prevalence of hypertension, dyslipidemia, and diabetes. Additionally, we found that the most prevalent frailty and pre-frailty clusters were “fatigue + poor mobility + low physical activity” and “poor mobility”, respectively. Overall, these results highlight a need to assess individual frailty characteristics within the Fried phenotype as the characteristics have individual and synergistic impacts on cardiometabolic outcomes.

### 4.1 Pre-Frailty and frailty prevalence

In the present study of PWH ≥ 50 years of age, the prevalence of pre-frailty was 45 %, which is similar to what was previously reported in CNICS (44 %) [10] and comparable to estimates reported among older PWH [12,18,19] and higher among HIV-uninfected persons [20]. Among PWH who were pre-frail, there was a greater burden of hypertension, dyslipidemia, and obesity. The prevalence of hypertension (61.8 %) among pre-frail PWH is higher than previous studies in pre-frail PWH [18,21]; however, it is similar to the prevalence of hypertension in the general older population [22]. These differences could be due to the way in which hypertension was ascertained within the different studies. Limited evidence exists on the prevalence of dyslipidemia among PWH who are pre-frail. However, the prevalence of dyslipidemia among PWH in the present study (65.8 %) was comparable to other clinic cohorts and the general population [[23], [24], [25], [26]]. Lastly, the prevalence of obesity (30.5 %) among PWH who were pre-frail was similar to that of previous studies [27].

Similarly, the prevalence of frailty in the present study was 16 %, which is comparable with previous reports in CNICS (13 %) [10] and other cohorts of PWH [28,29]. Among PWH who were frail there was a greater burden of hypertension, dyslipidemia, and diabetes. The prevalence of hypertension (62.6 %) among frail PWH was similar to that found in previous studies of frailty and HIV [30,31]. The prevalence of dyslipidemia (58.8 %) among frail PWH was slightly lower than what has been previously reported [[23], [24], [25], [26]]. The prevalence of diabetes mellitus (31.4 %) was higher than a nationally representative US sample of PWH receiving medical care [32]. The differences may reflect the burden of undiagnosed diabetes in the comparator study measured with fasting blood glucose and HBA1c.

### 4.2 Measurement of frailty clusters

There is not yet a consensus on the best approach to measuring frailty [33]. One such measure is the frailty index, which is based on accumulation of deficits [33,34], used among PWH predicted survival and incident multimorbidity [35]. The most commonly used approach in PWH, the Fried phenotype, has been modified in several ways. For example, this phenotype has been modified and validated in CNICS using 4 of the 5 characteristics used in the present study. Previous studies have focused on a 3-level frailty outcome variable based on the number of positive characteristics (robust, pre-frail, and frail); thus, to our knowledge, this was the first study to look at the clustering of frailty characteristics in PWH.

Many of the pre-frailty clusters were associated with a higher prevalence of cardiometabolic outcomes, the pre-frailty cluster with the highest prevalence of cardiometabolic conditions was the “poor mobility + low physical activity” cluster. This cluster had higher occurrences of all cardiometabolic outcomes compared to robust PWH. Similarly, we found significant relationships between frailty clusters and cardiometabolic outcomes. The specific cluster with the greatest prevalence was the “fatigue + poor mobility + low physical activity” cluster. The components underlying the two most prevalent frailty and pre-frailty clusters – poor mobility and low physical activity – both have independent associations with cardiometabolic disease in older adults. In previous work by others, it was discovered that poor mobility, indicated by slow walking, may have a neurologic etiology indicative of cognitive impairment [36,37]. As such, neurological degeneration may play an important role in the physical decline. Previous works have shown that poor mobility has been associated with cerebrovascular changes in the older adults [38]. In the I-Lan Longitudinal Aging Study (ILAS) cohort, poor mobility has been associated with cerebrovascular disease, hypertension and diabetes [37]. Additionally, the risks of engaging in low physical activity are well-established [7,39,40]. However, PWH have lower physical activity fitness levels and tend to not engage in regular physical activity when compared with other vulnerable populations [41]. Previous work in CNICS found that when compared with individuals who reported high levels of physical activity, individuals reporting very low physical activity had a nearly 2-fold greater risk of CVD [7]. These authors also found an increased risk of triglycerides, obesity, hypertension, and diabetes in PWH reporting very low levels of physical activity [7].

Fatigue is one of the most common symptoms of HIV, though it is underreported in PWH [42,43]. Fatigue has been strongly associated with anxiety and depression as a result of stressful events among PWH [[44], [45], [46]]. Little is known of the physiological correlates of fatigue as it relates to cardiometabolic health. Lastly, unintentional weight loss among PWH is multifactorial, and causes may include multiple determinants of health as well as complications of and therapies for the HIV infection [47]. Previous studies among seronegative individuals have found unintentional weight loss to be associated with a 50 % increase in CVD mortality [48].

Previous studies have shown that frailty, based on the Fried phenotype, is associated with the risk of chronic diseases in the general population and among PWH [31,49,50]. Yet, little evidence exists on whether effect modification exists by unique clusters of the frailty phenotype among PWH. While there is much utility for count-based frailty assessment in both clinical and community settings, we provide evidence that indicates that varying prevalence rates of cardiometabolic diseases exist within unique frailty clusters. Our results show that while PWH may be frail or pre-frail, the prevalence of cardiometabolic diseases may hold dynamic implications that need to be considered during intervention developments and routine clinical assessment. One interesting result was recorded among those with cerebrovascular disease. We found that using the count-based system, there was 1.7 times the prevalence of cerebrovascular diseases among pre-frail PWH when compared with robust PWH. However, within the same group of pre-frail PWH, there was 3 times the prevalence of cerebrovascular disease among the cluster of “poor mobility + low physical activity”, while another pre-frail subgroup had 2.4 times the prevalence for cerebrovascular diseases compared with the robust group. These findings have implications for identification of cardiometabolic disease within frailty categories and can aid in intervention development to prevent the transition from pre-frailty to frailty among PWH.

Our findings are subject to limitations. The cross-sectional nature of the present study limits causal inferences regarding the relations between frailty clusters and cardiometabolic disease and does not address how frailty progresses or recovers over time. CNICS may not generalize to individuals who do not yet know they have HIV or PWH who are not in clinical care. Secondly, we did not consider the links between the different cardiometabolic conditions, as we were primarily concerned with the prevalence of the cardiometabolic conditions within each frailty category. Additionally, CNICS uses a self-report PRO assessment. However, the PROs in CNICS demonstrates quality data capture and jointly reduces patient burden [51]. The PRO assessment in this study was only available for PWH who spoke English and Spanish, though it has been expanded to other languages. Additionally, our analyses included assessment from 2011 through 2020 and may be impacted by pandemic-related changes in clinical care and/or the frailty phenotypic components. Lastly, clustering the frailty characteristics limits the sample size within each cluster.

Despite the limitations, the present study benefited from several strengths, including a large, diverse cohort of PWH in the current era of HIV treatment. CNICS includes comprehensive clinical data and physician adjudicated outcomes. These findings highlight more detailed frailty subgroups of PWH and the prevalence of cardiometabolic disease and provides the foundation for future work in this area. Additional scientific exploration that determines the longitudinal impact of these clusters on clinical outcomes, as well as strategies to implement for reduction in frailty and cardiometabolic risk in individuals aging with HIV.

## 5 Conclusions

This study evaluated the prevalence of unique frailty clusters and the differential prevalence of cardiometabolic disease within unique frailty clusters. As the clinical presentation of frailty in older PWH is heterogeneous, it is important to understand how each frailty component affects health outcomes. This study demonstrated that the frailty components, independently and synergistically, are associated with increased prevalence of cardiometabolic disease. Additional research should focus on incorporating unique clusters into the overall assessment of frailty in routine care. The clinical care of frailty among PWH should be directed to managing the different components of frailty, as each of them may potentially be treatable. The incorporation and understanding of the frailty clusters into the routine care of aging PWH would allow for a unique screening for frailty and risk of cardiometabolic outcomes or vice versa. In addition to determining best practices for integrating the frailty clusters into clinical settings, future work should focus on the impact of these clusters on polypharmacy and mortality. Assessing the clustering of the frailty phenotypic components in routine care could be beneficial for risk stratification and reducing adverse health outcomes.

## Sponsor role

Sponsors had no role in the design of this study and no role in its execution, analysis, interpretation, or decision to submit the findings.

## Author contributions

RJ, EE, SR, AW & TB contributed to the concept and design. EE and RJ analyzed the data. RJ, SR, EE, HC, JACD interpreted the data. RJ and EE drafted the manuscript. RJ, EE, MK, RM, JJ, MK, MG, JF, SN, GB, JACD, HC, AW, & TB reviewed the manuscript critically, for important intellectual content.

## Financial disclosure

The National Institute of Allergy and Infectious Diseases (NIAID) grants R24AI067039, P30AI027757, P30AI050410, and P30AI027767, National Heart, Lung, and Blood Institute (NHLBI) grants K12HL143958, and R01HL126538, National Institute of Child Health and Human Development (NICHD) grant T32HD071866, and 10.13039/100000049National Institute on Aging (NIA) grant K01AG086063 supported this work. Its contents are solely the responsibility of the authors and do not necessarily represent the official views of the NIH.

## Declaration of competing interest

The authors declare no known conflicts of interest.

## Appendix Supplementary materials

Image, application 1
