# Supplementary material for: Cardiometabolic disease among frailty phenotype clusters in adults aging with HIV
Source: J Frailty Aging. 2025 Mar 8;14(2):100011. doi: 10.1016/j.tjfa.2025.100011 (PMC12183949; doi:10.1016/j.tjfa.2025.100011)
Supplement: Supplementary file 1 [file mmc1.docx]

| Supplemental Table 1: Poisson regression models for the association between cardiometabolic diseases and frailty overall and by clusters | | | | | | | |
| --- | --- | --- | --- | --- | --- | --- | --- |
|  | **Cerebrovascular disease** | **Cardiovascular disease** | **Diabetes Mellitus** | **Chronic Kidney Disease** | **Hypertension** | **Dyslipidemia** | **Obesity** |
|  | **PR, 95% CI** | | | | | | |
| Pre-frail vs. Robust | **1.74 (1.17 – 2.58)** | **1.57 (1.22 – 2.00)** | **1.43 (1.27 – 1.59)** | 1.03 (0.90 – 1.19) | **1.12 (1.06 – 1.18)** | **1.06 (1.02 – 1.11)** | **1.31 (1.18 – 1.45)** |
| Pre-frailty clusters vs. Robust group | | | | | | | |
| **Pre-frail (n)** | 70 | 163 | 600 | 432 | 1336 | 1424 | 633 |
| Fatigue + Weight Loss | 1.35 (0.33 - 5.50) | 0.75 (0.24 - 2.29) | 0.74 (0.44 - 1.26) | 0.72 (0.42 – 1.21) | 0.97 (0.79 – 1.18) | 0.93 (0.78 - 1.12) | 0.62 (0.36 - 1.07) |
| Fatigue + Poor mobility | **2.23 (1.01 - 4.91)** | 0.86 (0.41 - 1.82) | **1.76 (1.41 - 2.21)** | 0.79 (0.56 – 1.12) | 1.09 (0.96 – 1.23) | 1.05 (0.93 - 1.17) | **1.66 (1.35 - 2.05)** |
| Fatigue + Low Physical Activity | - | 1.70 (0.93 - 3.11) | **1.67 (1.29 - 2.17)** | 1.26 (0.93 – 1.72) | 1.08 (0.93 – 1.25) | 1.13 (1.00 - 1.27) | **1.73 (1.38 - 2.18)** |
| Weight Loss + Poor mobility | 2.42 (0.96 - 6.08) | **2.81 (1.68 - 4.71)** | **1.52 (1.13 - 2.02)** | 1.03 (0.71 – 1.49) | **1.24 (1.09 – 1.41)** | 1.01 (0.88 - 1.17) | 1.13 (0.82 - 1.56) |
| Weight Loss + Low Physical Activity | 0.80 (0.11 - 5.82) | 0.89 (0.29 - 2.73) | 1.45 (0.99 - 2.11) | 0.98 (0.59 – 1.63) | 0.98 (0.79 – 1.21) | 1.03 (0.86 - 1.23) | 1.23 (0.85 - 1.79) |
| Poor mobility + Low Physical Activity | **3.00 (1.72 - 5.22)** | **2.62 (1.85 - 3.72)** | **1.97 (1.67 - 2.32)** | 1.14 (0.91 – 1.41) | **1.27 (1.17 – 1.38)** | **1.12 (1.04 - 1.21)** | **1.51 (1.27 - 1.80)** |
| Fatigue | 1.56 (0.70 - 3.47) | **1.65 (1.04 - 2.63)** | 1.12 (0.88 - 1.44) | 1.06 (0.81 – 1.37) | 1.04 (0.93 – 1.17) | **1.11 (1.02 - 1.22)** | 1.06 (0.84 - 1.34) |
| Weight Loss | 1.26 (0.50 - 3.17) | 1.43 (0.84 - 2.42) | 0.99 (0.75 - 1.31) | 0.94 (0.70 – 1.27) | 0.97 (0.85 – 1.10) | 0.99 (0.88 - 1.10) | 0.95 (0.73 - 1.23) |
| Poor mobility | 1.65 (0.91 - 3.00) | 1.24 (0.81 - 1.89) | **1.37 (1.15 - 1.64)** | 0.96 (0.78 – 1.19) | **1.12 (1.04 – 1.22)** | 1.02 (0.94 - 1.10) | **1.38 (1.17 - 1.61)** |
| Low Physical Activity | 1.62 (0.83 - 3.15) | 1.28 (0.82 - 1.99) | **1.36 (1.12 - 1.65)** | 1.18 (0.96 – 1.45) | **1.18 (1.09 – 1.28)** | **1.11 (1.03 - 1.20)** | **1.35 (1.14 - 1.61)** |
|  | | | | | | | |
| Frail vs. Robust | 1.67 (1.00 – 2.79) | **2.05 (1.53 – 2.75)** | **1.64 (1.43 – 1.89)** | 1.02 (0.84 – 1.23) | **1.15 (1.08 – 1.23)** | 0.97 (0.91 – 1.04) | 1.14 (0.99 – 1.32) |
| Frailty clusters vs. Robust group | | | | | | | |
| **Frail (n)** | 23 | 73 | 239 | 147 | 477 | 448 | 197 |
| Fatigue + Weight Loss + Poor mobility + Low Physical Activity | 1.16 (0.42 - 3.20) | **2.61 (1.71 - 3.99)** | **1.61 (1.29 - 2.02)** | 1.25 (0.96 – 1.61) | **1.19 (1.07 – 1.33)** | 0.96 (0.85 - 1.08) | 0.99 (0.76 - 1.30) |
| Fatigue + Weight Loss + Poor mobility | 1.74 (0.54 - 5.56) | 0.89 (0.33 - 2.36) | **1.62 (1.19 - 2.21)** | 0.94 (0.61 – 1.47) | 1.01 (0.84 – 1.22) | 0.78 (0.63 - 0.96) | 0.81 (0.53 - 1.25) |
| Fatigue + Weight Loss + Low Physical Activity | 1.55 (0.38 - 6.32) | **2.85 (1.55 - 5.25)** | **1.50 (1.03 - 2.18)** | 0.71 (0.38 – 1.32) | 1.16 (0.97 – 1.38) | 1.10 (0.93 - 1.29) | 0.79 (0.48 - 1.28) |
| Fatigue + Poor mobility + Low Physical Activity | 1.51 (0.71 - 3.20) | **1.86 (1.21 - 2.84)** | **1.74 (1.45 - 2.10)** | 0.97 (0.76 – 1.24) | **1.19 (1.08 – 1.30)** | 1.03 (0.94 - 1.13) | **1.46 (1.22 - 1.74)** |
| Weight Loss + Poor mobility + Low Physical Activity | **2.91 (1.24 - 6.82)** | **2.07 (1.14 - 3.73)** | **1.55 (1.15 - 2.09)** | 0.99 (0.68 – 1.43) | 1.10 (0.95 – 1.28) | 0.92 (0.78 - 1.09) | 1.10 (0.79 - 1.53) |
| PR: Prevalence Ratio, 95% CI: Confidence Interval; Obesity missing n = 196; CKD missing n = 3; Model adjusted for age and sex | | | | | | | |

| Supplemental Table 2: Poisson regression models for the association between cardiometabolic diseases and frailty overall and by clusters | |
| --- | --- |
|  | **Multimorbidity** |
|  | **PR, 95% CI** |
| Pre-frail vs. Robust | **1.20 (1.19 – 1.41)** |
| Pre-frailty clusters vs. Robust group | |
| **Pre-frail (n)** |  |
| Fatigue + Weight Loss | 0.70 (0.46 – 1.06) |
| Fatigue + Poor mobility | **1.40 (1.16 – 1.68)** |
| Fatigue + Low Physical Activity | **1.51 (1.23 – 1.86)** |
| Weight Loss + Poor mobility | **1.53 (1.25 – 1.86)** |
| Weight Loss + Low Physical Activity | 1.16 (0.83 – 1.62) |
| Poor mobility + Low Physical Activity | **1.57 (1.37 – 1.78)** |
| Fatigue | 1.12 (0.93 – 1.35) |
| Weight Loss | 1.04 (0.84 – 1.28) |
| Poor mobility | **1.22 (1.07 – 1.40)** |
| Low Physical Activity | **1.38 (1.21 – 1.58)** |
| Frail vs. Robust | **1.26 (1.13 – 1.41)** |
| Frailty clusters vs. Robust group | |
| **Frail (n)** |  |
| Fatigue + Weight Loss + Poor mobility + Low Physical Activity | **1.26 (1.05 – 1.52)** |
| Fatigue + Weight Loss + Poor mobility | 0.97 (0.70 – 1.34) |
| Fatigue + Weight Loss + Low Physical Activity | 1.32 (0.98 – 1.77) |
| Fatigue + Poor mobility + Low Physical Activity | **1.33 (1.15 – 1.55)** |
| Weight Loss + Poor mobility + Low Physical Activity | 1.28 (1.00 – 1.63) |
| PR: Prevalence Ratio, 95% CI: Confidence Interval; Obesity missing n = 196; CKD missing n = 3; Model adjusted for age and sex | |

| Supplemental Table 3: Poisson regression models for the association between cardiometabolic diseases and frailty overall and by clusters | | | | | | | |
| --- | --- | --- | --- | --- | --- | --- | --- |
|  | **Cerebrovascular disease** | **Cardiovascular disease** | **Diabetes Mellitus** | **Chronic Kidney Disease** | **Hypertension** | **Dyslipidemia** | **Obesity** |
|  | **PR, 95% CI** | | | | | | |
| Pre-frail vs. Robust | **1.75 (1.17 – 2.62)** | **1.56 (1.21 – 2.02)** | **1.42 (1.25 – 1.62)** | 1.03 (0.90 – 1.19) | **1.12 (1.03 – 1.21)** | 1.06 (0.98 – 1.15) | **1.32 (1.17 – 1.49)** |
| Pre-frailty clusters vs. Robust group | | | | | | | |
| **Pre-frail (n)** | 70 | 163 | 600 | 432 | 1336 | 1424 | 633 |
| Fatigue + Weight Loss | **-** | 0.76 (0.25 - 2.29) | 0.74 (0.44 - 1.25) | 0.72 (0.42 - 1.22) | 0.95 (0.78 - 1.17) | 0.92 (0.76 - 1.10) | 0.64 (0.38 - 1.10) |
| Fatigue + Poor mobility | **-** | 0.84 (0.39 - 1.78) | **1.76 (1.40 - 2.20)** | 0.78 (0.55 - 1.11) | 1.09 (0.96 - 1.24) | 1.06 (0.94 - 1.18) | **1.69 (1.37 - 2.09)** |
| Fatigue + Low Physical Activity | **-** | 1.73 (0.95 - 3.15) | **1.66 (1.28 - 2.16)** | 1.27 (0.93 - 1.72) | **1.08 (0.93 - 1.25)** | 1.12 (0.99 - 1.26) | **1.71 (1.37 - 2.14)** |
| Weight Loss + Poor mobility | **-** | **2.79 (1.66 - 4.69)** | **1.51 (1.13 - 2.02)** | 1.04 (0.72 - 1.50) | **1.25 (1.10 - 1.41)** | 1.02 (0.89 - 1.18) | 1.18 (0.86 - 1.62) |
| Weight Loss + Low Physical Activity | **-** | 0.86 (0.28 - 2.65) | 1.44 (0.99 - 2.09) | 0.98 (0.59 - 1.62) | 0.98 (0.79 - 1.21) | 1.04 (0.87 - 1.23) | 1.29 (0.89 - 1.86) |
| Poor mobility + Low Physical Activity | **-** | **2.57 (1.81 - 3.65)** | **1.96 (1.66 - 2.31)** | 1.13 (0.91 - 1.41) | **1.27 (1.17 - 1.38)** | **1.13 (1.05 - 1.22)** | **1.56 (1.32 - 1.85)** |
| Fatigue | **-** | **1.70 (1.07 - 2.70)** | 1.12 (0.87 - 1.44) | 1.07 (0.82 - 1.38) | 1.04 (0.93 - 1.16) | **1.10 (1.00 - 1.20)** | 1.05 (0.84 - 1.33) |
| Weight Loss | **-** | 1.42 (0.84 - 2.39) | 0.99 (0.75 - 1.31) | 0.94 (0.70 - 1.27) | 0.97 (0.86 - 1.11) | 1.00 (0.90 - 1.11) | 0.98 (0.76 - 1.27) |
| Poor mobility | **-** | 1.24 (0.82 - 1.89) | **1.37 (1.15 - 1.64)** | 0.96 (0.78 - 1.19) | **1.12 (1.04 - 1.22)** | 1.02 (0.94 - 1.10) | **1.35 (1.15 - 1.59)** |
| Low Physical Activity | **-** | 1.29 (0.83 - 2.00) | **1.36 (1.12 - 1.64)** | 1.19 (0.97 - 1.46) | **1.18 (1.09 - 1.28)** | **1.11 (1.03 - 1.19)** | **1.35 (1.14 - 1.61)** |
|  | | | | | | | |
| Frail vs. Robust | 1.68 (0.99 – 2.84) | **2.02 (1.48 – 2.75)** | **1.64 (1.40 – 1.93)** | 1.02 (0.84 – 1.24) | **1.16 (1.04 – 1.29)** | 0.98 (0.88 – 1.09) | **1.19 (1.00 – 1.41)** |
| Frailty clusters vs. Robust group | | | | | | | |
| **Frail (n)** | 23 | 73 | 239 | 147 | 477 | 448 | 197 |
| Fatigue + Weight Loss + Poor mobility + Low Physical Activity | **-** | **2.53 (1.65 - 3.89)** | **1.61 (1.28 - 2.02)** | 1.25 (0.97 - 1.63) | **1.20 (1.08 - 1.34)** | 0.97 (0.86 - 1.10) | 1.05 (0.80 - 1.37) |
| Fatigue + Weight Loss + Poor mobility | **-** | 0.87 (0.33 - 2.30) | **1.63 (1.20 - 2.21)** | 0.95 (0.61 - 1.49) | 1.03 (0.85 - 1.24) | 0.79 (0.64 - 0.98) | 0.87 (0.57 - 1.33) |
| Fatigue + Weight Loss + Low Physical Activity | **-** | **2.71 (1.45 - 5.04)** | **1.49 (1.02 - 2.16)** | 0.69 (0.37 - 1.30) | 1.16 (0.98 - 1.38) | 1.11 (0.94 - 1.32) | 0.81 (0.50 - 1.31) |
| Fatigue + Poor mobility + Low Physical Activity | **-** | **1.85 (1.21 - 2.84)** | **1.74 (1.45 - 2.10)** | 0.98 (0.77 - 1.25) | **1.19 (1.09 - 1.31)** | 1.04 (0.95 - 1.14) | **1.52 (1.27 - 1.81)** |
| Weight Loss + Poor mobility + Low Physical Activity | **-** | **2.08 (1.16 - 3.74)** | **1.55 (1.15 - 2.10)** | 0.99 (0.68 - 1.44) | 1.10 (0.95 - 1.28) | 0.91 (0.77 - 1.07) | 1.08 (0.78 - 1.50) |
| PR: Prevalence Ratio, 95% CI: Confidence Interval; Obesity missing n = 196; CKD missing n = 3, CD4 cell count missing n = 5; Model adjusted for age, sex, ART status and CD4 cell count | | | | | | | |

| Supplemental Table 4: Poisson regression models for the association between cardiometabolic diseases and frailty overall and by clusters | |
| --- | --- |
|  | **Multimorbidity** |
|  | **PR, 95% CI** |
| Pre-frailty clusters vs. Robust group | |
| **Pre-frail** | **1.30 (1.17 – 1.45)** |
| Fatigue + Weight Loss | 0.70 (0.47 - 1.06) |
| Fatigue + Poor mobility | **1.41 (1.17 - 1.69)** |
| Fatigue + Low Physical Activity | **1.51 (1.23 - 1.85)** |
| Weight Loss + Poor mobility | **1.56 (1.27 - 1.90)** |
| Weight Loss + Low Physical Activity | 1.17 (0.84 - 1.62) |
| Poor mobility + Low Physical Activity | **1.57 (1.38 - 1.80)** |
| Fatigue | 1.12 (0.93 - 1.35) |
| Weight Loss | 1.05 (0.85 - 1.29) |
| Poor mobility | **1.22 (1.07 - 1.40)** |
| Low Physical Activity | **1.38 (1.21 - 1.58)** |
| Frail vs. Robust | **1.28 (1.11 – 1.48)** |
| Frailty clusters vs. Robust group | |
| **Frail** | **1.28 (1.11 – 1.48)** |
| Fatigue + Weight Loss + Poor mobility + Low Physical Activity | **1.29 (1.07 - 1.55)** |
| Fatigue + Weight Loss + Poor mobility | 1.00 (0.72 - 1.39) |
| Fatigue + Weight Loss + Low Physical Activity | 1.32 (0.98 - 1.78) |
| Fatigue + Poor mobility + Low Physical Activity | **1.36 (1.17 - 1.59)** |
| Weight Loss + Poor mobility + Low Physical Activity | **1.28 (1.00 - 1.63)** |
| PR: Prevalence Ratio, 95% CI: Confidence Interval; Obesity missing n = 196; CKD missing n = 3, CD4 cell count n = 5; Model adjusted for age, sex, ART status and CD4 cell count | |
